# Ten tips on management of BK nephropathy in kidney transplant patients

**DOI:** 10.1093/ckj/sfag061

**Published:** 2026-02-20

**Authors:** Colin C Geddes, Paul J Phelan

**Affiliations:** Glasgow Renal and Transplant Unit, Queen Elizabeth University Hospital Glasgow, Glasgow, UK; Renal Unit, Royal Infirmary of Edinburgh, Edinburgh, UK

**Keywords:** acute rejection, BK viraemia, BK virus–associated nephropathy, immunosuppression, kidney transplantation

## Abstract

Management of BK virus after kidney transplantation remains challenging, as recently published updated guidelines identify ongoing gaps in the evidence. In this article we present tips for management based on evidence and clinical experience. Testing blood for BK virus by polymerase chain reaction should be part of the workup for any patient with significant deterioration in kidney transplant function. All kidney transplant biopsies should be tested by immunohistochemistry for the presence of SV40 large T antigen if there are features of tubulointerstitial inflammation on light microscopy. A transplant kidney biopsy that does not include medulla does not exclude BK virus–associated nephropathy (BKVAN). Tubulointerstitial inflammation with positive SV40 staining and undetectable BK virus in the blood implies a different polyomavirus nephropathy such as JC virus. Clinicians should consider the evidence carefully before deciding whether or not to adopt a BK viraemia screening strategy in their unit. If a strategy of screening for BK viraemia in the absence of transplant dysfunction is adopted, then it makes sense to target the first year and for screening to be at least every 4 weeks. The optimal reduction in immunosuppression in response to BK viraemia or BKVAN has not been established by clinical trials. A rapid decline in BK viral load after reduction in immunosuppression appears to be a strong risk factor for impending T cell–mediated rejection. Clinical trials of the addition of potentially effective anti-polyomavirus agents including leflunomide, fluoroquinolones, intravenous immunoglobulin concentrate, cidofovir and statins have not shown benefit. BK viraemia or BKVAN recurrence after viral clearance is rare.

## INTRODUCTION

BK virus–associated nephropathy (BKVAN) presents a challenge for patients and clinicians after kidney transplant. The BK polyomavirus (BKV), first isolated in 1971, is a latent virus that reactivates under immunosuppression, particularly following kidney transplantation. BK viraemia occurs in ≈10–20% of kidney transplant recipients, mostly in the first year. Biopsy-proven BKVAN develops after viraemia in 1–10% of all kidney transplants, with graft loss attributed to BKVAN reported in 30–40% of affected patients [1]. Risk factors for BK viraemia and BKVAN include older age, male recipient sex, use of tacrolimus (versus ciclosporin), use of higher corticosteroid doses and recipient BKV antibody negativity if the donor is BK antibody positive [1].

BKVAN is suspected in patients with otherwise unexplained deterioration in transplant function and detectable BK virus DNA in peripheral blood. The diagnosis is confirmed by kidney biopsy. Increasingly clinicians may elect not to perform a biopsy if significant viraemia is detected via screening, as the treatment is similar to that of established BKVAN. In resource-limited settings, microscopy of fresh urine for characteristic decoy cells may be helpful for diagnosing BKVAN, but sensitivity is low [2].

BKVAN management relies primarily on immunosuppression reduction guided by blood viral load kinetics and graft function. No specific treatment strategy has yet proven effective in randomised controlled trials (RCTs). The management of BKVAN has come into focus in the last year with the publication of the Second International Consensus Guidelines on the Management of BK Polyomavirus in Kidney Transplantation, the UK Guideline on Management of BK Polyomavirus Infection and Disease Following Kidney Transplantation and a Cochrane review of interventions for BK virus infection in kidney transplant recipients [1, 3, 4]. Despite these guidelines, there remains uncertainty around the merits and frequency of screening for BK viraemia, the optimal intervention once BK viraemia is detected and the treatment of BKVAN.

This article presents 10 practical tips on the management of BKVAN aimed at clinicians managing patients in the kidney transplant clinic. The tips are based on the recently published guidelines and a literature search for relevant recently published and ongoing clinical trials. We have also drawn on our clinical experience as transplant nephrologists—one from a centre that adopts a BK viraemia screening protocol and the other from a centre that does not.

Testing blood for BK virus by polymerase chain reaction (PCR) should be part of the workup for any patient where there has been deterioration in kidney transplant function.

It is important to be aware that only a minority of patients who have detectable BK virus in blood will go on to develop BKVAN. There is evidence that a viral load of ≥10 000 copies/ml has a high positive predictive value for BKVAN [1, 3] and therefore should prompt action such as kidney transplant biopsy or a reduction in immunosuppression. If the first detectable viral load is <10 000 copies/ml and kidney function is not deteriorating rapidly, it is reasonable to repeat a blood test for BK viral load and graft function in 1–2 weeks. If the viral load is decreasing at that stage, then continued monitoring without change in medication is reasonable. If the viral load is increasing rapidly, then action such as kidney transplant biopsy or a reduction in immunosuppression is appropriate.

All kidney transplant biopsies should be tested by immunohistochemistry for the presence of SV40 large T antigen if there are features of tubulointerstitial inflammation on light microscopy.

SV40 is a stain that is specific for polyomaviruses and staining in regions of inflammation is diagnostic of polyomavirus nephropathy. It is often not possible to distinguish the inflammation of BKVAN from the inflammation of T cell–mediated rejection without SV40 staining. In our centres, all indication transplant biopsies are stained for SV40.

A transplant kidney biopsy that does not include medulla does not exclude BKVAN.

BKVAN is best seen in the medulla of the kidney and can sometimes be absent in the cortex but present in medulla [5].

Tubulointerstitial inflammation with positive SV40 staining and undetectable BK virus in the blood implies a different polyomavirus nephropathy such as JC virus [6, 7].

JC virus can be identified on kidney biopsy using techniques such as immunohistochemistry staining with JC-specific antibodies, *in situ* hybridization or molecular testing. JC virus DNA can also be identified in peripheral blood. JC virus nephropathy is rare, poorly studied and it is less clear that the temporal sequence of viruria followed by viraemia followed by nephropathy seen in BKVAN also applies to JC virus nephropathy [6, 7]. Management of JC virus nephropathy should probably follow the same principles as BKVAN although this is based on evidence from a few case reports only [6].

Clinicians should consider the evidence carefully before deciding whether or not to adopt a BK viraemia screening strategy in their unit.

BK viraemia screening strategies post-transplant remain controversial. ‘Frequent’ screening of blood to detect BK virus replication and intervene by immunosuppression reduction in advance of the development of BKVAN as recommended by clinical guidelines [1, 3, 4] seems like a good idea. However, it is important that patients and clinicians are aware that BK virus screening has not been subjected to RCTs and carries the risk of rejection following a reduction in immunosuppression in response to BK viraemia. This is acknowledged in the second international guideline and the UK consensus guideline [1, 3]. In contrast, the authors of the recently published Cochrane review of interventions for BK virus infection in kidney transplant recipients stated, ‘We are confident that intensive screening for BK virus prevents the loss of the transplanted kidney’ [4]. However, their confidence was based on only one study that was published 20 years ago only in abstract form [8]. The abstract describes a single-centre ‘cluster randomisation’ trial of de novo kidney transplant recipients and contained a very limited description of a BK screening method that was based initially on urine testing. The graft survival in the 648 patients who had BK virus screening was 100%, compared with 75% in the 260 patients who did not have screening [8]. The imbalance in numbers makes it unlikely that this was an RCT and the 100% survival in the intervention group raises questions about follow-up. Without further details, it is not possible to draw any firm conclusions and we do not regard this as evidence of effectiveness of BK screening that informs contemporary management.

If a strategy of screening for BK viraemia in the absence of transplant dysfunction is adopted, then it makes sense to target the first year after transplant and for screening to be at least every 4 weeks.

This is based on the known kinetics of BK viraemia: new BK viraemia and BKVAN are rare after 1 year and clinically meaningful increases in BK viral load can take as little as 4 weeks [1]. We agree with the schedule suggested by the Second International Consensus Guidelines of screening kidney transplant recipients for plasma BK virus load monthly until month 9, then every 3 months until 2 years post-transplantation [1].

The optimal reduction in immunosuppression in response to BK viraemia or BKVAN has not been established by RCTs.

A reasonable stepwise approach is to initially halve the dose of antimetabolite and aim for calcineurin inhibitor trough blood concentrations at the lower end of the target range. It is important to be aware that a decrease in BK viral load and stabilisation or improvement in transplant function is expected to take weeks or months rather than days, so patience is needed. It is stated in the guidelines that if viral load has not decreased by 10-fold by 4 weeks, then further reductions in immunosuppression are advised. In our experience the viral load sometimes increases initially before starting to decrease by 4 weeks, so extra patience may be needed. Patients should be aware that studies of immunosuppression reduction in response to BKVAN or viraemia report 10–30% subsequent incidence of acute rejection [1]. In our experience, the incidence of acute rejection following immunosuppression reduction is lower than that. Observational studies, *in vitro* studies and secondary analyses of RCTs suggested conversion to mammalian target of rapamycin (mTOR) inhibitors might be associated with a reduced incidence of BKVAN and more rapid clearance of BK viraemia than reduction in mycophenolate. However, a recently published RCT using everolimus to address that question concluded that was not the case [9] and we do not recommend a switch to mTOR inhibitor instead of antimetabolite reduction.

A very rapid decline in BK viral load after reduction in immunosuppression appears to be a strong risk factor for impending T cell–mediated rejection.

We have observed this very occasionally in cases of lymphocyte repopulation after anti-thymocyte globulin or alemtuzumab induction where a dramatic decrease in viral load has coincided with lymphocyte repopulation and heralded acute rejection. It therefore makes sense to monitor BK viral load and kidney function frequently after reducing immunosuppression (e.g. every 2 weeks) and consider increasing immunosuppression again if there is a very rapid decrease in viral load, especially if other factors suggest a higher-than-normal risk of immunological rejection.

Clinical trials of the addition of potentially effective anti-polyomavirus agents including leflunomide, fluoroquinolones, intravenous immunoglobulin (IVIG) concentrate, cidofovir and statins have not shown benefit [1, 3, 4].

Other potential therapies are in development. The results of a completed phase 3 trial (NCT05769582) of a neutralising monoclonal antibody against BKV are awaited after promising results in a first-in-human trial [10]. Viral-specific T cell therapy has shown promise in phase 1 and 2 clinical trials in kidney transplant recipients with BK viraemia [11, 12] but no phase 3 trial is registered as yet.

It is understandable that clinicians and patients may want to try additional treatments if viral load is persistently >10 000 copies/ml after immunosuppression reduction, especially if there is proven BKVAN with deteriorating transplant function. Under these circumstances, administration of IVIG probably has the least weak evidence base [13]. IVIG preparations contain BK virus–neutralising antibodies and small observational studies suggest improved BKV clearance compared with historic controls [1]. The optimal dose is unknown. A dose of 0.1–0.3 g/kg body weight once every 2–4 weeks with monitoring of BK viral load seems reasonable. There are no published RCTs of IVIG used to treat BK viraemia or BKVAN. We have also used IVIG for rare cases of coexistent BKVAN and acute rejection.

BK viraemia or BKVAN recurrence after viral clearance is rare.

BK viraemia or BKVAN is said to recur in 10–20% of cases following viral clearance after immunosuppression reduction [1], but in our experience the incidence is lower than that and is rarely of clinical significance. Patients at moderate or high long-term rejection risk can therefore have maintenance immunosuppression increased again, although generally not back to the initial immunosuppression at the time of initial BK viraemia. We would monitor for recurrence of BK viraemia for several months after doing this. In addition, failure of a kidney transplant due to BKVAN is not a contraindication to further transplant [1], although it seems sensible to wait until BKV has cleared from the blood before further transplant.

## CONCLUSION AND FUTURE DIRECTIONS

These practical tips address some of the uncertainties that make management of BK virus after kidney transplant one of the most challenging situations for clinicians. We anticipate the field will advance significantly in the next few years. Measurement of donor and recipient BKV neutralising antibody composition at the time of transplant has the potential to refine risk stratification [14]. Improved understanding of BK virus pathogenicity in relation to signalling pathways and micro-RNAs in recent years will hopefully open new avenues to develop specific therapies [15–17]. It is likely that modification of immunosuppression load will remain the main focus of management of BK viraemia and BKVAN. It is hoped that improved clinical tools to quantify immunosuppression will translate into improved ability for clinicians to individualise the balance between minimising rejection risk and avoiding infective complications such as BKVAN.

## Data Availability

No new data were generated or analysed in support of this research.
